# A Hematological Travesty in the Setting of Alcohol Use Disorder and Liver Cirrhosis

**DOI:** 10.7759/cureus.19096

**Published:** 2021-10-28

**Authors:** Ryan F Amidon, Christ Ordookhanian, Elizabeth D Liu, Omar Abdallah, Paul Kaloostian

**Affiliations:** 1 Medicine, Medical College of Wisconsin, Milwaukee, USA; 2 Medicine, University of California, Riverside, Riverside, USA; 3 Biochemistry, University of California, Riverside, Riverside, USA; 4 Neurological Surgery, Riverside Community Hospital, Riverside, USA; 5 Neurological Surgery, Paul Kaloostian M.D. Inc., Riverside, USA

**Keywords:** alcohol-related liver disease, intraparenchymal hemorrhage, ethanol induced, binge drinking, platelet count (plt), liver cirrhosis

## Abstract

Excessive ethanol consumption is associated with an increased risk of developing health complications, especially in individuals with pre-existing thrombocytopenia and cirrhosis. Here, we describe a case of a patient with a history of alcoholic cirrhosis, hypertension, and thrombocytopenia, presenting with significant decline following an incidence of binge drinking. Radiography identified a large non-lobar intraparenchymal hemorrhage. However, due to severe thrombocytopenia that was unresponsive to platelet therapy, the possibility of pursuing any form of surgical intervention was negated. Surgical contraindication and a lack of response to subsequent medical management contributed to the family’s decision to opt for conservative medical treatment and comfort care. This case showcases the potential for liver cirrhosis in the setting of chronic alcohol use disorder to pave the way for terminal intracerebral hemorrhage.

## Introduction

Despite accounting for approximately 4% of worldwide deaths, ethanol consumption remains an exceptionally prevalent hobby around the world [1]. Excessive alcohol intake is the third leading preventable cause of mortality and liver cirrhosis, present in 10% to 20% of heavy drinkers, and accounts for about 17% of alcohol-related deaths [1-3]. About 24% of teenagers and young adults reported binge drinking on weekends in one study, although other studies report up to a 50% prevalence of binge drinking in their samples [2,4]. Heavy alcohol use can wreak havoc on virtually every organ system, but it most notably causes serious damage to the liver. A study of 6,366 individuals without baseline liver disease showed that binge drinking elevated the risk of developing the liver disease [5]. Alcohol-induced liver disease may lead to other health complications, including cirrhosis, simple steatosis, steatonecrosis, and steatohepatitis [3,6].

Cirrhosis represents a late stage of hepatic fibrosis and subsequent hepatic functional decline that occurs from a chronic disease of the liver, including nonalcoholic fatty liver disease, chronic alcohol consumption, hemochromatosis, and chronic viral hepatitis [7]. Such diseases can generate abundant damage to healthy hepatocytes, overwhelming cellular repair capabilities thus leading to the production of excess fibrous connective tissue and scarring of the liver, which ultimately leads to the decline in hepatic biochemical processes. As scarring worsens, liver insult may not be amenable to reversal. Hepatic transplantation may become necessary in advanced pathology. Cirrhosis represents a growing public health issue, affecting approximately 0.27% of adults in the United States, and its prevalence has been projected to increase in the future [8]. More alarmingly, 69% of adults diagnosed with cirrhosis reported being unaware they possessed liver disease [8]. This is unfortunate due to the prospects of undesirable outcomes, demonstrated by cirrhosis labeled as the eighth leading cause of mortality in the United States in 2010 [9].

Chronic ethanol ingestion, binge drinking, and cirrhosis are notorious risk factors for stroke. Although ischemic strokes account for 80% to 90% of instances of alcohol-related strokes, intracranial and subarachnoid hemorrhages represent 10% to 20% of cases [10]. Intracranial hemorrhage incidence is about 24.6 per 100,000 person-years, with rates increasing with age as evident by a 10-fold increase in adults over 85 years old when compared to adults between the ages of 45 and 54 years [10]. Following an intracranial hemorrhage, the prognosis may be rather unfortunate, with 30-day mortality rates as high as 45% in admitted patients [10]. Despite the introduction of new management strategies, the high associated mortality rate has not seen significant improvements. Unfortunately, only 12% to 39% of patients who survive reacquire functional independence [11].

Here, we present a patient with major risk factors for hemorrhagic stroke. Following an episode of binge drinking, the patient was found with neurological compromise. Neuroimaging revealed an acute intraparenchymal hemorrhage (IPH); however, the patient’s low platelet count contraindicated surgical intervention. Despite efforts to improve platelet levels, medical management was ineffective, leaving comfort care as the primary management option.

## Case presentation

Following an episode of binge drinking, a 50-year-old male with a history of liver cirrhosis, hypertension, and thrombocytopenia was discovered with right-sided weakness and altered mental status. Prior to being discovered, the patient spent several days in his car, likely contributing to the low alcohol levels detected in his system. Laboratory studies revealed diminished fibrinogen and platelet levels, both indicators of major bleeding risk (Table 1).

**Table 1:** Hematology results from laboratory studies.

Laboratory test	Emergency department admission values	Day one in hospital values	Reference values
White blood cell count	6.2 × 10^9^/L	6.9 × 10^9^/L	3.4-9.6 × 10^9^/L
Red blood cell count	3.59 × 10^12^/L	3.67 × 10^12^/L	4.35-5.65 × 10^12^/L
Hemoglobin	11.0 g/dL	11.2 g/dL	13.2-16.6 g/dL
Hematocrit	32.1%	34.3%	38.3-48.6%
Mean corpuscular volume	92.1 fL	93.0 fL	80-100 fL
Mean corpuscular hemoglobin	29.9 pg/cell	30.1 pg/cell	25.4-35.6 pg/cell
Mean corpuscular hemoglobin concentration	31.7 Hb/cell	31.6 Hb/cell	31-36 Hb/cell
Red cell distribution width	16.4%	16.9%	11.9-15.5%
Platelet count	43 × 10^9^/L	47 × 10^9^/L	150-400 × 10^9^/L
Neutrophils (%)	86.5	91.1	54-62
Lymphocytes (%)	9.5	6.2	25-33
Monocytes (%)	3.4	2.1	3-7
Eosinophils (%)	0.3	0.2	1-3
Basophils (%)	0.3	0.4	0-1
Prothrombin time	12.7 s	12.5 s	11-15 s
Partial thromboplastin time	26.1 s	27.0 s	25-40 s
International normalized ratio	1.2	1.3	<1.1
Fibrinogen	177 mg/dL	179 mg/dL	200-400 mg/dL

**Figure 1:**
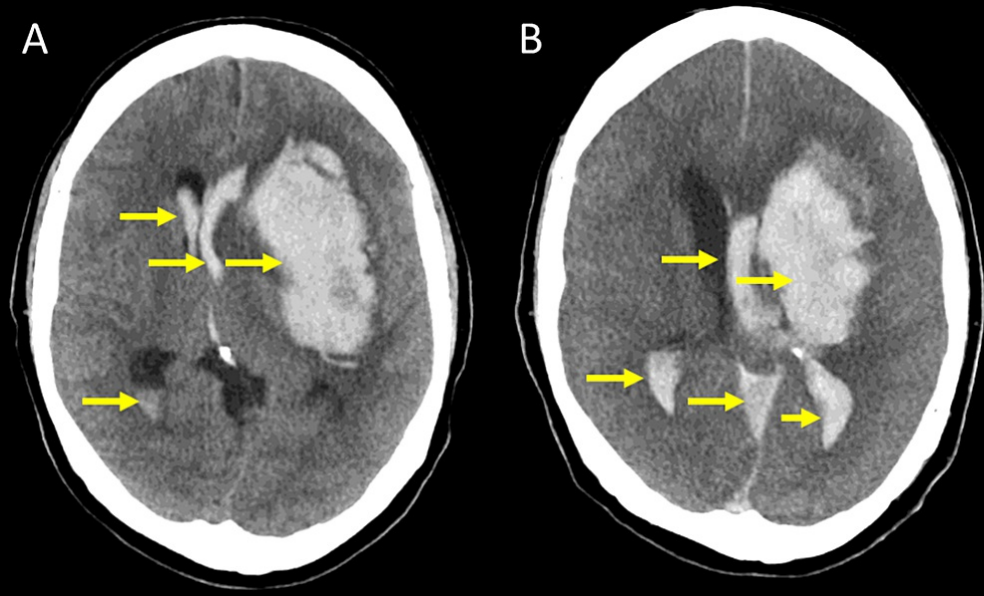
(A) Initial axial computed tomography (CT) scan taken after the presentation to the emergency department. (B) A follow-up axial CT scan of the head taken five days later demonstrates minute differences, even after medical management.

**Figure 2:**
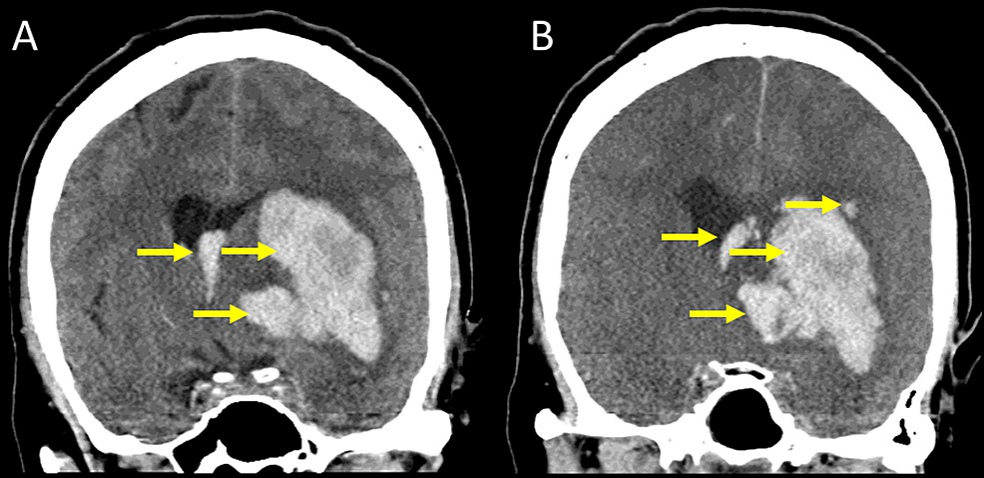
(A) Initial computed tomography (CT) scan taken in the coronal plane after the presentation to the emergency department. (B) A follow-up coronal CT scan of the head taken in the coronal plane five days later demonstrates minute differences, even after medical management.

Studies also revealed a high red cell distribution width (RDW), likely due to anemia of chronic disease, as suggested by a normal mean corpuscular volume (MCV) indicating normocytic anemia. With the patient’s increased risk of bleeding, their normocytic anemia was likely to develop into microcytic anemia. Lymphocyte count was pseudo-low, likely stemming from the high neutrophil count brought on by an underlying infection. The low platelet count resulted from pre-existing, severe thrombocytopenia, with no significant signs of improvements between tests. In conjunction with low levels of fibrinogen, which primarily functions in blood clot formation, this low platelet count demonstrated this patient’s high risk of experiencing a bleeding event. Computed tomography (CT) scans of the brain were performed upon admission to the emergency department, revealing an acute intraparenchymal hemorrhage (IPH) with a 90-cc clot located in the basal ganglia and a 1-cm midline shift, which could not be significantly improved five days later as revealed by follow-up CT scans (Figures 1, 2).

Although medical management was pursued with mannitol and 3% hypertonic saline to lower intracranial pressure, improvements were negligible.

Given the poor hematology test results and increased risk of bleeding events, our patient was an unsuitable surgical candidate. To raise the platelet count to a sufficiently high level necessary for surgical candidacy, five platelet transfusions were provided. Despite these transfusions, there were only minor improvements, rising from 43,000/μL to 47,000/μL, and counts remained dangerously low to perform any intervention safely. As a result, our patient stayed under intubation and was managed conservatively pending next of kin directions. With no neurological improvement, continued lack of alertness or orientation, and inability to follow commands, the prognosis was discussed with the patient’s family and hospice was endorsed.

## Discussion

Intraparenchymal hemorrhages (IPHs) are categorized as lobar or non-lobar. Lobar IPH bleeding occurs within the cortex and non-lobar IPH bleeding occurs within the brainstem, basal ganglia, deep cerebellum, or thalamus [12]. IPHs are caused by a wide range of etiologies. Tumors, cerebral amyloid angiopathy, thrombosis of dural venous sinuses and cortical veins, ischemic stroke with hemorrhagic conversion, arteriovenous fistulas, arteriovenous malformations, vasculitis and vascular malformations, venous angiomas, and aneurysms are all potential causes of IPH [13]. Additionally, ruptured Charcot-Bouchard aneurysms in the basal ganglia, cerebellum, pons, and thalamus, all regions with plentiful small penetrating vessels, are also well-known causes of IPH [10]. It is postulated that lipohyalinosis of small arterioles, resulting from chronic hypertension, can induce Charcot-Bouchard aneurysm ruptures [13].

Hypertensive persons have up to nine times the risk of undergoing intracerebral hemorrhage compared to those without hypertension [14], and unfortunately, hypertension is a common chronic condition, affecting 34% to 59% of males and 33% to 52% of females who are 40 to 79 years old [15]. Other risk factors of IPH include alcohol consumption, diabetes, increased high-density lipoprotein cholesterol, low total cholesterol, low non-high-density lipoprotein cholesterol, smoking, and anticoagulant therapy [12,13]. An increased risk of developing IPH is associated with excessive alcohol consumption, as well as platelet dysfunction, coagulopathy, and endothelial injury [12].

Alcohol intake can prompt the development of alcoholic cirrhosis, which may result in ascites, variceal hemorrhage, hepatic encephalopathy, spontaneous bacterial peritonitis, hepatorenal syndrome, hepatopulmonary syndrome, and hepatocellular carcinoma, all signs of decompensated cirrhosis [16]. Alcoholic cirrhosis may lead to serious, irreversible health conditions, including decompensation and liver cancer, even with abstinence. The absence of decompensated cirrhosis symptoms represents the compensated form, which is not as severe and is associated with an average survival rate of more than 12 years [17]. Alcohol consumption, bleeding, infection, dehydration, constipation, and medications are known risk factors for the development of decompensated cirrhosis.

Thrombocytopenia, a condition characterized by low platelet count, is a common complication associated with liver disease and cirrhosis. There are numerous mechanisms via which thrombocytopenia can occur, including dysfunctional platelet production, portal hypertension, hypersplenism, and bone marrow suppression [18]. Normal platelet count is considered to be at least 150,000/μL, mild thrombocytopenia ranges from 100,000 to 150,000/μL, moderate thrombocytopenia ranges from 50,000 and 100,000/μL, and severe thrombocytopenia refers to levels under 50,000/μL. Mild thrombocytopenia is present in 75% of patients with chronic liver disease, while moderate thrombocytopenia occurs in about 13% of patients with cirrhosis [18]. Conversely, cirrhosis is also known to trigger a hypercoagulable state. Liver cirrhosis is also associated with abnormal aminotransferases, elevated serum bilirubin, increased alkaline phosphatase/gamma-glutamyl transpeptidase, heightened international normalized ratio (INR) and prothrombin time (PT), as well as hyponatremia [19].

When treating patients with intracerebral hemorrhage, it is critical to prioritize stabilization, survival, and minimization of secondary injury to the brain. When surgery is viable, and when indicated by the presence of large hematoma, clinical deterioration, or coma, the surgeon can perform decompression and extract clots while also removing toxic blood products and addressing potential complications from intracerebral hypertension if necessary [12,13]. Key indications for surgery include intracerebral hemorrhage with brainstem compression, hydrocephalus from obstructed ventricles, and neurological deterioration, especially in cases where the hemorrhage is cerebellar [20]. During the treatment process, maintaining optimal platelet count is necessary for invasive procedures. When thrombocytopenia is identified, the platelet count should be increased to the desired level using replacement therapy: at minimum 50,000/μL for moderate-risk procedures and about 100,000/μL for high-risk procedures and instances of active bleeding [16]. When platelet transfusions are ineffective at sufficiently raising the platelet count, alternative treatment options should be considered. The patient’s blood pressure should also be controlled to prevent further intracerebral hemorrhaging [20]. Acute intracerebral hemorrhage management commonly involves coagulation factor replacement therapy, blood pressure management, and surgery, but the appropriate combination of treatment methods will vary case-to-case to best suit the patient’s exact clinical context.

While our patient’s history of hypertension, binge drinking activity, liver cirrhosis, and thrombocytopenia may have all contributed to the development of non-lobar IPH, the low, unresponsive platelet count was the deciding factor in forgoing surgical intervention, and it was the main predictor of our patient’s prognosis. The odds of survival and functional recovery are already low in IPH patients, and with treatment options limited to medical management and comfort care, our patient’s prognosis was poor. Medical management was ineffective, leaving our patient’s comfort and quality of life our primary objective.

## Conclusions

Chronic alcoholism and binge drinking are likely to remain significant threats to global health. Binge drinking, particularly in cases with pre-existing hypertension, cirrhosis, and thrombocytopenia, increases the risk for large-volume intracerebral hemorrhaging. In this case, we describe a patient with intraparenchymal hemorrhage (IPH) likely brought on by a combination of binge drinking and an increased risk of hemorrhage due to prior history of several conditions. When medical management is ineffective, surgical intervention may be a viable option. However, with this patient, surgery was contraindicated due to low platelet counts and the lack of sufficient response to treatments aiming to increase platelet counts. As a result, the patient’s family was recommended comfort care. In cases with concurrent thrombocytopenia, multiple platelet transfusions are not always successful at raising platelet counts to the necessary value to safely qualify for invasive procedures. Platelet counts are critical determinants for surgical candidacy, and unfortunately, as in the case of our patient, the prognosis is grave when surgery is contraindicated due to low platelet counts. We hope to improve the public’s understanding of the correlation between alcohol consumption, liver disease, and stroke to promote healthier lifestyle choices and preventative healthcare efforts.
